# Supplementary figures and images for: The association between the use of video games, social media and online dating sites, and the symptoms of anxiety and/or depression in adults aged 25 and over
Source: Glob Ment Health (Camb). 2024 Jan 18;11:e11. doi: 10.1017/gmh.2024.2 (PMC10882175; doi:10.1017/gmh.2024.2)

**Supplementary Figure 1: Timeline of the TEMPO cohort study**

**
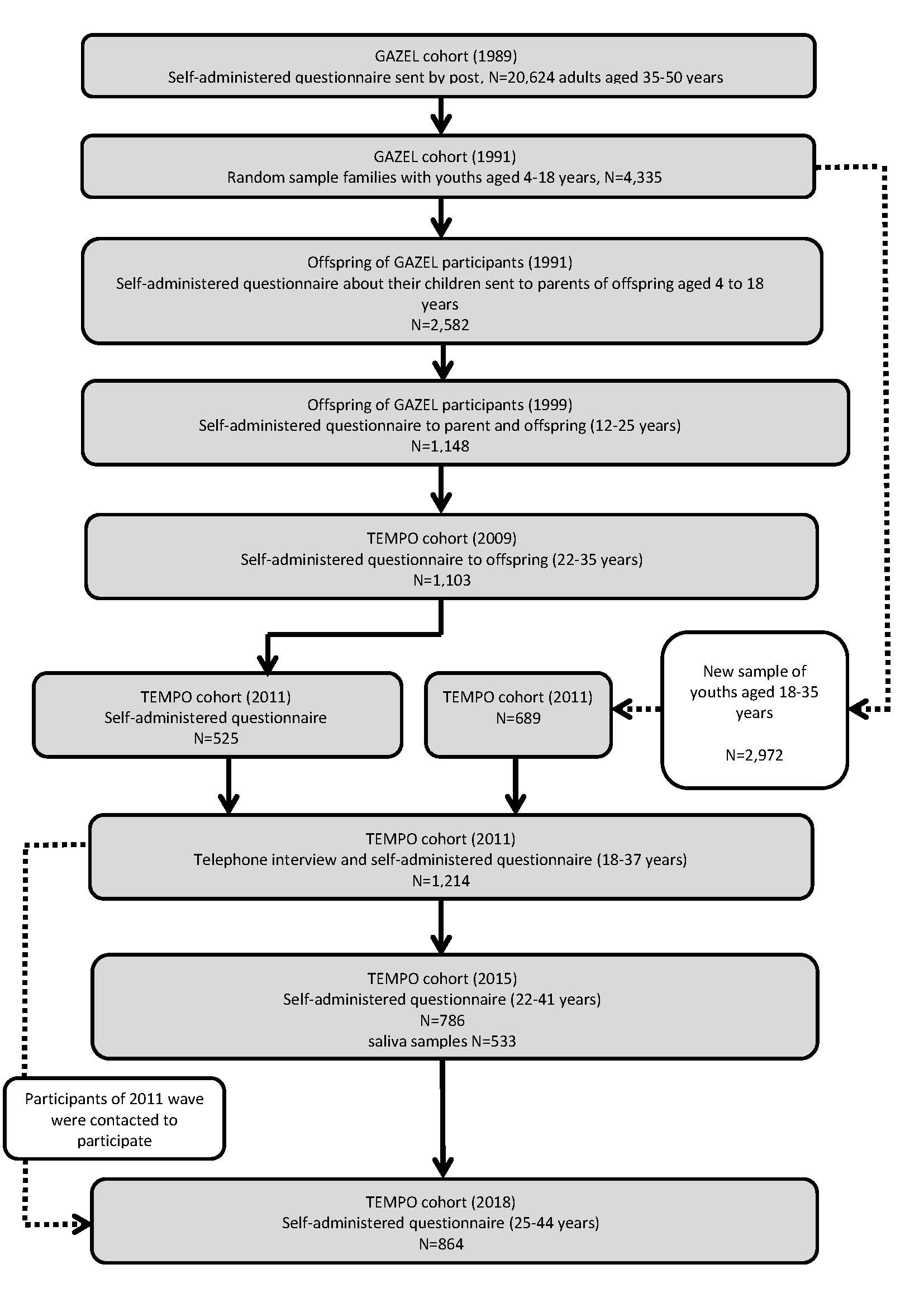
**

Supplement: El Haddad et al. supplementary material [file S2054425124000025sup001.docx]
